# Predictors of Depression in Caucasian Patients with Amyotrophic Lateral Sclerosis in Romania

**DOI:** 10.3390/brainsci10080470

**Published:** 2020-07-22

**Authors:** Motataianu Anca, Andone Sebastian, Radu Cristina, Bajko Zoltan, Barcutean Laura, Balasa Adrian, Voidazan Septimiu, Stoian Adina, Maier Smaranda

**Affiliations:** 1 Neurology 1 Clinic, Emergency Clinical County Hospital Mures, 540136 Targu Mures, Romania; motataianuanca@gmail.com (M.A.); adn.sebastian.007@gmail.com (A.S.); cristinag.radu@yahoo.com (R.C.); bzoltan2003@yahoo.com (B.Z.); laurabarcutean@gmail.com (B.L.); adina.stoian@umfst.ro (S.A.); maier_smaranda@yahoo.com (M.S.); 2 Neurology Department, University of Medicine, Pharmacy, Science and Technology, “G. E. Palade”, 54012 Targu Mures, Romania; 3 Neurosurgery Department, University of Medicine, Pharmacy, Science and Technology, “G. E. Palade”, 540142 Targu Mures, Romania; 4 Neurosurgery Clinic, Emergency Clinical County Hospital Mures, 540136 Targu Mures, Romania; 5 Epidemiology department, University of Medicine, Pharmacy, Science and Technology, “G. E. Palade”, 540142 Targu Mures, Romania; septi_26_07@yahoo.com; 6 Pathophysiology Department, University of Medicine, Pharmacy, Science and Technology, “G. E. Palade”, 540142 Targu Mures, Romania

**Keywords:** depression, amyotrophic lateral sclerosis, physical disability, autonomic symptoms, caregiver, spirituality

## Abstract

Depression remains an underdiagnosed comorbidity which significantly decreases the quality of life in amyotrophic lateral sclerosis (ALS) patients. We aimed to investigate the prevalence of depression in a cohort of ALS patients with more than one year of disease evolution. A total of 50 ALS patients were evaluated with the Beck Depression Inventory II (BDI-II) and cognition, using the Mini-Cog Standardized Instrument (MCSI). The clinical disability was evaluated using the ALS Functional Rating Scale (ALSFRS). The prevalence of depression was 42.8%. A lower BDI-II score was significantly correlated with a higher education level, the spouse as a caregiver, spiritual devotion, and employment status (*p* < 0.05). A multiple linear regression analysis between the BDI-II score as the dependent variable and various independent variables such as spirituality, caregiver status, educational level, and occupational status revealed that only the type of caregiver (spouse or parent/child) significantly affected the BDI-II total score (*p* = 0.006). The functional disability significantly correlated with loss of appetite and loss of libido (*p* < 0.001). A high education, spiritual devotion, high ALSFRS, and the presence of the spouse as the caregiver were associated with the absence of depression.

## 1. Introduction

Amyotrophic lateral sclerosis (ALS) is a devastating progressive disorder that destroys motor neurons (MN) in the motor cortex, brain stem, and spinal cord, resulting in muscle weakness and premature death, usually due to respiratory failure, within 2–5 years from the onset of the disease [1]. ALS has emerged as a multisystem disorder in which the clinical, pathological, and genetic features overcome the boundaries of a pure MN involvement, with both physical and psychological distress.

The neurodegenerative process starts in MN and spreads alongside the functional networks in the central nervous system [2]. Motor involvement dominates the clinical picture, but the extra motor areas involved in the degenerative process are being increasingly recognized because of the various non-motor symptoms, such as behavioral changes, cognitive impairment, and autonomic dysfunction [2,3,4]. The diagnosis of ALS casts a shadow upon the dreams, expectation, and life plans of patients [5].

Depression in patients with ALS is an under-recognized comorbidity and can be in part explained by the reserved prognosis of this diagnosis and because of the continuous motor function decay, with a progressively reduced quality of life (QoL) [4,6]. The prevalence of depression in ALS varies in different studies from very low (13%) to high values (up to 59%), depending on the assessment measure [6,7,8,9,10]. Depression carries harmful effects on both the QoL and survival of ALS patients, being independent of the physical impairment and thus associated with an increased rate of disease progression [11,12]. 

The aim of the present study was to prospectively investigate the prevalence of depressive symptoms in ALS patients and to characterize the impact of several factors such as physical disability, disease duration, and psychosocial variables upon depression, and the importance of autonomic symptoms on functional disability.

## 2. Materials and Methods

An observational, non-interventional, prospective study was established for the assessment of functional impairment and depressive symptoms in order to evaluate the interrelations between these variables in a Romanian cohort of ALS patients. We evaluated 50 ALS patients from the ALS outpatient and inpatient Department of Neurology, Mures County Clinical Emergency Hospital, Tirgu Mures, Romania, between July 2017 and May 2020.

The inclusion criteria were: (a) a diagnosis of sporadic ALS established by a neuromuscular physician as probable or definite ALS (according to the El Escorial revised criteria) [13]; (b) at least 12 months from the ALS diagnosis (in order to avoid the impact that this incurable diagnosis has upon the patient); (c) the absence of other chronic illnesses; (d) the patients having agreed to psychological and cognitive testing.

The exclusion criteria were: (a) patients with psychiatric diseases prior to the diagnosis of ALS; (b) a history of alcohol or drug abuse; (c) neurological disease (other than ALS); (d) patients with cognitive function impairment; (e) the presence of pseudobulbar signs as a confounder of depressive symptoms; (f) the presence of other systemic illnesses (neoplasia, orthopedic, cardiovascular, or respiratory pathologies), (g) patients on sedatives, antidepressants, anti-anxiety medication at enrolment; (h) patients who were new to our department and came only for the second opinion. Based on these exclusion criteria, 15 patients were excluded from the study group. All the patients underwent a complete clinical neurological examination.

For a neuropsychological assessment, all the patients completed the two standardized questionnaires: the Beck Depression Inventory II (BDI-II) and Mini-Cog standardized instrument (MCSI).

The MCSI, a standardized assessment of mental status, was administered in order to assess cognition in all the participants. The cognitive impairment was defined as an MCSI score of <3. The study patients with cognitive impairment were excluded [14]. 

BDI-II is a 21-item multiple-choice self-report scale and a standard questionnaire of depression. BDI-II, the Romanian version, was completed by all the study participants. BDI-II assesses the presence and severity of the depressive symptoms and evaluates the cognitive, motivational, autonomic, and somatic domains. The total score can range from 0 to 63, and the used cut-offs were: <10—no depressive symptoms; 10–13—minimal depressive symptoms; 14–19—mild depressive symptoms; 20–28—moderate depressive symptoms; ≥29—severe depressive symptoms [15]. The cut-off score of 14 was used to identify patients with significant depressive symptoms. We selected five questions (Q) from BDI-II in order to describe the autonomic symptoms: Q16. changes in sleep; Q17. tiredness or fatigue; Q18. changes in appetite; Q19. loss of weight; Q21. loss of sexual interest. The selected questions reflect the vegetative symptoms exhibited by the included patients. 

The patient’s physical impairment due to ALS was assessed by the Revised Amyotrophic Lateral Sclerosis Functional Rating Scale (ALSFRS), based on 12 questions, each rated from 0 (total loss of function) to 4 (normal) on a point scale. The grade of functional disability ranges from 0 (severe) to 48 (normal), therefore a lower score signifies a greater disability. ALSFRS evaluates the bulbar function (speaking, salivation, swallowing), motor function in the upper and lower limbs (handwriting, cutting food, handling utensils, dressing and hygiene, turning in bed, walking, climbing stairs) and respiratory functions (dyspnoea, orthopnoea, respiratory insufficiency). Additionally, ALSFRS is a useful tool for evaluating the activities of daily living in ALS patients [16]. 

The ALS clinical data were recorded based on the disease duration (time from the disease’s onset in months), time from the ALS diagnosis (in months), type of diagnosis (bulbar: ALS_arm and ALS_leg), use of ALS medication (Riluzol), and use of anti-depressive and antianxiety agents. The general socio-demographic factors were assessed by the standard patient history: age, gender, education, religion, marital status, number of children, main caregiver (spouse, children, parents), employment status, and perceived economic status. The educational levels were stratified into low (≤8 years of schooling) and high (≥9 years of education). The employment status was classified into employed (mental labor and physical labor) and retired (due to the age or due to ALS).

All the patients gave signed informed consent to the participation. The Research Ethics Board of the Clinical County Emergency Hospital Mures approved the current study protocol and it was performed following the principles stated in the Declaration of Helsinki (grant number 293/5/2020—from University of Medicine, Pharmacy, Sciences and Technology “George Emil Palade” Targu Mures). 

### Statistical Analyses

For the statistical analyses, we used the GraphPad 3,6 State Software, IBM SPSS Statistics v20, and Microsoft Excel 2010. The Shapiro–Wilk normality test was used for continuous variables (age, BDI-I score, ALSFRS score). The Student’s T test and ANOVA test were applied to evaluate the differences between the averages of two continuous variables (expressed as mean ± SEM − Std. error of mean), and for nonparametric variables we used the Mann–Whitney and Kruskall Wallis test (expressed as the median and minimum/maximum). A post-hoc analysis based on Dunn’s approach with a Bonferroni correction was also conducted to identify the source of difference. For the categorical variables, we used the Chi-square test, and in order to evaluate the correlation between the distributions of other variables we used Spearman’s rho test. The odds ratio (OR) was calculated in order to demonstrate the probability or susceptibility to depression. The scores obtained from the BDI application were used as a dependent variable, and the rest of the parameters mentioned above were considered independent variables. For a multivariate analysis, we coded the following variables: high risk of depression—1, no risk of depression—0. A statistical interpretation was performed against the statistical threshold of *p* = 0.05, and values of *p* below 0.05 were considered as statistically significant.

## 3. Results

### 3.1. Baseline Group Characteristics

The socio-demographic and clinical characteristics of the study group are summarized in Table 1. The patients’ average age (mean ± SEM) was 56.17 ± 1.68 years (with the age of onset between 29 and 75 years), the time (months) from the onset to diagnosis was 10.67 ± 0.69, the time (months) since diagnosis was 15.90 ± 1.12, and the disease duration (months) was 26.17 ± 3.22. The mean ALSFRS was 37.14 ± 1.16.

### 3.2. Depressive Symptoms in ALS Patients

Among the ALS patients, the univariate analysis between the clinical and demographic data considered as independent variables and the BDI-II score, as a dependent variable, indicated that the BDI-II score did not statistically significantly differ between males or females or in those with a low, medium, or high perceived economic level. In contrast, the patients with a higher educational level (high school or university), a spouse as a caregiver, spiritual devotion; who were employed or retired due to age; and with a higher ALSFRS (>30) had lower BDI-II scores than those with lower education, with their parents or children as caregivers, who were retired due to ALS, and with a lower ALSFRS (≤30) (all *p* < 0.05). (Table 2)

In our study group, depression was found in 42.8% of the ALS patients. In Table 3, a comparative analysis was performed using the statistical chi-square test for the demographical characteristics and the selected variables dependent on the presence or absence of depression. Therefore, this showed that ALS patients without depression had a significantly higher education level, spiritual devotion, a spouse as a caregiver, and lower functional disability (ALSFRS > 30) than those with depression (all *p* < 0.05, Table 3). The patients with depression had parents or children as caregivers (*p* < 0.0001). The patients with depression had a smaller period of time from onset to diagnosis (8.3 ± 1.98 months), while for the patients without depression the period of time from onset until diagnosis was longer (13.8 ± 4.14 months), the difference between the two groups being statistically significant (*p* < 0.01). There was no statistically significant difference between the two groups for disease duration (months).

Among the ALS patients, a multiple linear regression analysis was performed with the BDI-II score as the dependent variable, and religious devotion, caregiver, education level, and occupation status as independent variables. In this model, only the type of caregiver significantly affected the BDI-II total score (*p* = 0.006, Table 4).

A logistic regression was performed, with the dependent variable depression (BDI-II) and the caregiver status/ALSFRS as independent variables (Table 5). We identified the caregiver defined by children and parents as a risk factor (OR 48.54, CI 95%: 1.88–126.28) and the ALSFRS score as a protective factor (OR 0.58, CI 95% 0.37–0.91). In this model, the ALSFRS score is strongly associated with depression from a protective standpoint, while the caregiver status is associated with the risk of depression. 

### 3.3. Analysis of Autonomic Symptoms Domains from BDI-II in ALS Patients

Based on the five Qs we previously described from BDI-II’s autonomic domain (sleep, fatigue, appetite, weight loss, and sexual interest), the results of the exploratory correlational analyses showed that only appetite, weight loss, and sexual interest significantly correlated with functional disability by ALSFRS (Table 6). This shows that in ALS patients, physical disability has a lesser impact on appetite, weight loss, and sexual interest. 

Additionally, regarding Q21, we performed a statistical analysis based on age groups (<50 years old, 51–60 years old, 61–70 years old, and >70 years old) and we found no statistically significant differences (all *p* > 0.05). 

### 3.4. Analysis of ALS form and the Potential for Depression Induction

We performed a logistic regression with the dependent variable being depression and the independent variables being the type of ALS (bulbar and spinal: ALS_arm and ALS_leg) in the selected patients. In this model, the patient population with upper limb involvement had the lowest depression score as calculated by BDI-II (*p* = 0.040, OR < 1, CI 95% 0.279–0.973), compared to patients with bulbar or leg involvement (all *p* > 0.05). (Table 7).

## 4. Discussion

Depression has negative effects upon survival and QoL in ALS patients [8,12]. Depression may be an early sign of frontal lobe degeneration, and the symptoms of depression may also superimpose with the clinical signs of cognitive impairment [17]. In our study, we excluded patients with cognitive impairment and we found that the prevalence of depression in ALS patients was 42.8%. The reasoning behind this decision was based on the possible alteration of depression inventories, secondary to cognitive impairment; in patients with frontal lobe dysfunction, the depressive symptoms can be masked by anosodiaforia [18], with fronto-temporal dementia being frequently associated with ALS, in up to 40% of the cases [19,20]. The screening test used, MCSI, has both a high sensitivity and sensibility for the detection of general cognitive symptoms [21], but in order to accurately classify the area of cognitive decline, subsequent tests should be used [22]. Previously, researchers from the Karolinska Institute in Sweden described that in a large cohort of ALS patients (no. 1752), during the first year after an ALS diagnosis the risk for developing depression was 6.7-fold higher than in the control group [11]. Recently, Sandstedt et al. (2018) and Jakobsson et al. (2017) reported in two observational and longitudinal studies that depression seems to decrease over time after an ALS diagnosis [23,24]. Our results, at more than one year after the initial diagnosis, revealed that the depression is still significantly present in ALS patients. 

The prevalence of depression in ALS varies significantly across the studies, according to the research assessment tools used and the variability of the patient population. It was demonstrated that the occurrence of depression is more probable after an ALS diagnosis, more likely within one year after the onset of motor symptoms [11,25]. The prevalence of depression in our study is within the range reported by other studies [26,27]. The possible explanation for this high prevalence of depression at more than one year after ALS diagnosis is due to severe emotional and psychological distress when facing a no hope diagnosis, and on the other hand because of the physical disabilities that worsen over time. Other studies on the depression prevalence in ALS included patients on anti-depressive treatments, and this can be a possible explanation for a lower rate of depression in those patients [6]. 

Another aspect regarding the presence and evaluation of depression in ALS patients has to consider the anti-depressive properties of the agent of choice—riluzole. Its effects vary, with a wide range of symptoms, having glutamatergic properties but also both antiepileptic and neuroprotective effects, being used as an off-label medication for mood disorders. The pharmacokinetics behind the anxiolytic effect may be partly explained by the action upon the glutamatergic system, but also on the release of dopamine and norepinephrine [28,29]. No effects have been noted regarding the serotonin pathways, a neurotransmitter actively involved in the mechanism of depression [30]. A number of clinical trials targeting depression support the theory that riluzole carries certain anti-depressive and anxiolytic properties, being used for bipolar disorders, major depression, and anxiety disorders [31,32,33,34]. However, considering the aforementioned aspects, it may be premature to suppose that riluzole actively impacted the depression prevalence, given that the synaptic disruption is continuous throughout the evolution of the disease [35]. More pharmacokinetic studies are required in order to assess the long-term effects of riluzole regarding depression. 

We found a strong association between depression and the severity of functional disability, as indicated by ALSFRS, but the results from other studies regarding the association between depression and functional disability are controversial. Atassi et al. (2011) found no correlation between depression and functional status, as measured by ALSFRS and the respiratory status of these patients [6]. Chen et al. (2014) and Sandstedt et al. (2018) demonstrated in Chinese and Swedish ALS patients, respectively, that depression was not associated with physical disability [23,26]. On the contrary, Oh et al. (2012) and Korner et al. (2015) found in groups of ALS patients from South Korea and from Germany, respectively, that it increased physical disability, as assessed by ALSFRS, and was positively correlated with depressive symptoms [9,36]. Wei et al. (2016) demonstrated that depression was correlated with increased physical disability in ALSFRS, but the depression had no impact on the progression or survival of ALS patients in a Chinese population study [37]. The influence that the functional disability has on depression might be explained by the fact that decreasing levels of independence limit the working ability, therefore distressing the social life and in the end, leading to a significant decrease in the patients’ self-esteem. Our study reported that the patients with predominantly upper limb involvement, rather than a lower limb deficit or the bulbar form of ALS, tend to present lesser depressive symptoms [9]. The impact of ambulatory loss has been associated with a higher depression index [38], but a direct correlation between the time of onset and the physical burden has to be taken into account. Our patients had at least one year of disease evolution, and a longer disease duration allows the patients to adapt coping mechanisms for their own disabilities. 

Caregivers play an essential role in the life of ALS patients. Studies have demonstrated that the caregivers often develop psychological distress symptoms themselves. In our study, we found that patients who are assisted by their parents or children are more likely to have depressive symptoms than patients who are cared for by their partners (spouse). This finding can probably be related to the feelings of worthlessness or guilt and due to putting the patient’s own health and well-being above all others and by neglecting their lives and health, which can determine the feeling of being a caregiver burden. Emotional processing difficulties about the feeling of being a burden may be a factor in developing depressive symptoms in patients cared for by children or parents [39,40,41]. Lillo et al. (2012) identified that the caregiver burden in ALS patients is associated with psychological and behavioral changes, rather than the physical disability alone [42]. Mitchell et al., in an extensive study following individuals with various neurological disabilities that are being taken care of in a home setting, reported that more than half of ALS patients are married, compared to patients with traumatic brain injury, epilepsy, and stroke. Another important aspect underlined by the authors was that, for ALS patients, the caregivers live together with the patients. A higher distress level was identified in the caregivers of ALS patients, notably due to the fact that, among all the neurological pathologies that require assistance, ALS caregivers are more thoroughly involved in the activities of daily living [43]. The choice for a spouse as a primary caregiver is secondary to them, usually being the most important familial bond [44].

The burden experienced by ALS caregivers is important, and recently Grabler et al. (2018) found that in ALS, the caregivers of patients affected by depression are more likely to mirror their patients’ mindsets and feelings. These findings draw attention to the fact that psychological support for ALS caregivers should start sooner [45]. 

In our patient study group, the presence of depression was associated with a lower educational level. Lule et al. (2008) reported that, in German ALS patients, depression was negatively correlated with the educational status of the patients [46]. On the contrary, Chen et al. (2015) demonstrated in Chinese patients that depression was not correlated with the educational level [26]. Differences in cultural and socio-economic backgrounds, as well as different assessment tools for depression, can partially explain the discrepancies among the studies. 

Spirituality is accepted as a superior human dimension which significantly contributes to the well-being and health [47]. It is recognized as an essential domain of QoL in patients faced with incurable illnesses, and also in the end-of-life care for these patients. In our study group, we found significantly lower BDI-II scores in ALS patients with religious devotion. There are little data in the literature regarding spirituality care in ALS patients. Dal Bello-Haas et al. (2000) reported that religious well-being may have a positive impact on the QoL in ALS patients [48]. Additionally, O’Brien et al. (2015) reported on 137 ALS patients that religious faith sustains and helps patients in avoiding desperation and aids in discovering life’s meaning after the diagnosis of ALS [49]. The impact of ALS diagnosis affects more than just the patient whose body it inhabits. It also affects the patient’s mental, emotional, and spiritual plan, and therefore spiritual assistance might aid and positively influence the well-being of the patient. 

Our study showed that factors such as age, gender, employment, perceived economic status, marital status, and the use of Riluzole were not correlated with depression. These results are in accordance with previous studies [6,50,51,52]. 

There are limited data about autonomic dysfunction symptoms in ALS patients. In our study, we found that the loss of appetite, loss of weight, and loss of sexual interest significantly correlated with functional disability, as evaluated by ALSFRS. A lower body mass index and weight loss are recognized as negative prognostic factors for survival in ALS patients [53,54]. Körner et al. (2013) reported that weight loss is a frequent and serious problem and is not solely due to dysphagia [55]. In a previous study, it was observed that autonomic symptoms are relatively common in ALS patients, and male erectile dysfunction was frequently described, together with gastrointestinal and sudomotor abnormalities [56]. 

Recently, Sandstedt et al. (2018) reported in a three-year observational study that depression, anxiety, and fatigue coexisted over time in ALS patients and were not related with the disease severity. The constant impairment was fatigue, without any variation throughout the study [23]. A survey study by Nicholson et al. (2018) reported that fatigue was the most prevalent and untreated symptom in ALS patients [57]. These results are in contrast with ours, mainly due to the method used. The fatigue was evaluated based on specific fatigability questionnaires, while in our case we based our results on the data extrapolated from the BDI-II autonomic domain. Many factors, including physiological (pain, sleep disturbances) and psychological (depression, loss of internal motivation) problems might contribute to fatigue in ALS patients.

Our study has several limitations. The first is represented by the small sample size, but this seems to be common for ALS research, because of its low prevalence (worldwide all-age 4.5/100.000 individuals) and low incidence (worldwide all-age incidence 0.78 per 100.000 person-year/Europe incidence 1.89/100.000 person-year) [58,59]. The second limitation is due to the use of MCSI for cognitive evaluation, but this test cannot evaluate frontotemporal dementia, which is the most common form of cognitive impairment in ALS patients. Creating a validation cohort has proved to be one of the most significant hardships of this present study due to the low incidence of ALS. Another important aspect is the presumed anti-depressive effects of riluzole, with it being unclear if the long-term use of this agent actively impacts the presence or intensity of depressive symptoms.

Based on these findings, depression cannot be explained by motor and functional impairment alone. Patients’ personality traits such as adaptability, together with feelings of self-esteem and both social and spiritual support, might influence the acceptance of this devastating diagnosis. 

## 5. Conclusions

The prevalence of depression in ALS is high. A low education level, the absence of spiritual devotion, a high physical disability, and having children or parents as caregivers were associated with depression. Despite the absence of a curative treatment for ALS, maintaining patients’ well-being and their adequate QoL is very important. The active monitoring and treatment of depression in ALS patients can alleviate the burden of the disease. Knowing this aspect, a holistic approach can be valuable for patient care, and this knowledge can contribute to a more patient-focused treatment.

## Figures and Tables

**Table 1:** Clinical and demographic characteristic of the study patient group.

Variable		Cases (Total = 35)
Sex	Male	21 (60%)
	Female	14 (40%)
Age	<50	10 (28.57%)
	50–65	21 (60%)
	>65	4 (11.42%)
Occupation	Physical work employee	8 (22.85%)
	Intellectual work employee	4 (11.42%)
	Illness retirement (ALS)	12 (34.28%)
	Age retirement	11 (31.42%)
Married	Yes	31 (88.57%)
	No	4 (11.42%)
Has children	Yes	29 (82.85%)
	No	6 (17.14%)
Self-perceived economical level	Low	7 (20%)
	Medium	24 (68.57%)
	High	4 (11.42%)
ALS type	Bulbar	3 (8.57%)
	Limb	32 (91.42%)
Caregiver	Husband/Wife	19 (54.28%)
	Parents/Children	16 (45.71%)
Education level	Elementary school degree	8 (22.85%)
	High school degree	20 (57.14%)
	University degree	7 (20%)
Religious/Spiritual person	Yes	24 (68.57%)
	No	11 (31.42%)
Demographic environment	Rural	16 (45.71%)
	Urban	19 (54.28%)
ALS-FRS groups	≤30	10 (28.57%)
	>30	25 (71.42%)
Antidepressants treatment	Yes	10 (28.57%)
	No	25 (71.42%)
Rilutek treatment	Yes	27 (77.14%)
	No	8 (22.85%)

**Table 2:** Univariate analysis of associations between the socio-demographic and clinical variables and patients’ Beck Depression Inventory II (BDI-II) scores.

Related Factors	N	Mean ± SEM	*p*-Value
Sex			0.185 *
Male	21	13.14 ± 1.82	
Female	14	18 ± 3.46	
Education level			0.014 *
Elementary school degree	8	19.25 ± 3.19	
High school degree	20	16.25 ± 2.58	
University degree	7	7.00 ± 1.02	
Religious/Spiritual person			0.003 *
Yes	24	11.71 ± 1.56	
No	11	22.45 ± 3.74	
Caregiver			0.0002 *
Husband/Wife	19	9.53 ± 1.17	
Parents/Children	16	21.69 ± 2.89	
Occupation			0.043 **
Physical work employee	8	14.50 ± 2.29	
Intellectual work employee	4	5.00 ± 0.40	
Illness retirement (ALS)	12	20.83 ± 3.88	
Age retirement	11	12.91 ± 2.38	
Self-perceived economical level			0.25 **
Low	7	13.00 ± 2.60	
Medium	24	16.88 ± 2.39	
High	4	8.00 ± 1.47	
ALSFRS			0.0001 *
≤30	10	26.30 ± 3.50	
>30	25	10.60 ± 1.32	

* Student Test, ** ANOVA test and Bonferroni’s multiple comparisons test.

**Table 3:** Comparative analysis of patients’ subgroups, according to the presence or absence of significant depressive symptoms.

Variable	With Depression	Without Depression	*p*-Value *
Male:Female	7:8	14:6	0.16
Age groups			0.28
<50	5	5	
50–65	7	14	
>65	3	1	
Education level			0.0001
Elementary school degree	7	1	
High school degree	8	12	
University degree	0	7	
Self-perceived economical level			0.19
Low	3	4	
Medium	12	12	
High	0	4	
Religious Devotion/Spirituality			0.016
Yes:No	7:8	17:3	
Caregiver			0.0001
Husband/Wife	2	17	
Parents/Children	13	3	
ALS-FRS groups			0.0001
≤30	9	0	
>30	6	20	
Married			0.65
Yes:No	13:2	18:2	
Has children			0.69
Yes:No	12:3	17:3	
Demographic environment			0.43
Rural	8	8	
Urban	7	12	
Occupation			0.065
Physical work employee	4	4	
Intellectual work employee	0	4	
Illness retirement (ALS)	8	4	
Age retirement	3	8	
Riluzol treatment			0.64
Yes:No	11:4	16:4	

* Chi-square test.

**Table 4:** Multivariate linear regression analysis for the independent predictors of patients’ BDI-II scores.

Variables	Coefficient	*p*-Value	Odds Ratio	95% CI	
				Lower	Upper
Religious/Spiritual person	1.749	0.251	5.754	0.289	114.282
Caregiver	3.493	0.006	32.903	2.673	405.051
Education level	1.579	0.237	4.852	0.354	66.493
Occupation	0.836	0.593	2.307	0.108	49.396

**Table 5:** Logistic regression analysis for the independent predictors of patients’ BDI-II scores.

Variable	Coefficient	Odds Ratio	95% CI	*p*-Value
Caregiver	6.19144	48.54	1.88 to 126.28	0.0290
ALSFRS	−0.53067	0.58	0.37 to 0.91	0.0198

**Table 6:** Correlation between autonomic symptoms subscores/questions and Amyotrophic Lateral Sclerosis Functional Rating Scale (ALSFRS).

Question (Q)	ALSFRS	
	Coefficient	*p*-Value
Q16 Sleep	−0.291	0.090
Q17 Fatigue	−0.315	0.065
Q18 Appetite	−0.710	<0.001
Q19 Weight loss	−0.403	0.016
Q21 Sexual interest	−0.620	<0.001

**Table 7:** Logistic regression between the (BDI-II) scores and ALS type.

Variable	Coefficient	Odds Ratio	95% CI	*p*
ALS_bulbar	−0.28377	0.7529	0.4923 to 1.1516	0.1905
ALS_arm	−0.65110	0.5215	0.2793 to 0.9735	0.0409
ALS_leg	0.092727	1.0972	0.6517 to 1.8471	0.7271
